# Protective Effects of Red Ginseng Oil against Aβ_25–35_-Induced Neuronal Apoptosis and Inflammation in PC12 Cells

**DOI:** 10.3390/ijms18102218

**Published:** 2017-10-23

**Authors:** Seonah Lee, Kumju Youn, Woo-Sik Jeong, Chi-Tang Ho, Mira Jun

**Affiliations:** 1 Department of Food Science and Nutrition, Dong-A University, 37, Nakdong-daero 550 beon-gil, Saha-gu, Busan 49315, Korea; seonah_lee@daum.net (S.L.); kjyoun@dau.ac.kr (K.Y.); 2 Department of Food & Life Science, College of Biomedical Science & Engineering, Inje University, 197, Inje-ro, Gimhae-si, Gyeongsangnam-do 50834, Korea; jeongws@inje.ac.kr; 3 Department of Food Science, Rutgers University, New Brunswick, NJ 08901, USA; ho@aesop.rutgers.edu

**Keywords:** Alzheimer’s disease, β-amyloid peptide, red ginseng oil, apoptosis, inflammation

## Abstract

One of pathological characteristics of Alzheimer’s disease (AD), aggregation and deposition of β amyloid (Aβ), has been accepted as a potent activator of neuronal cell death. Red ginseng is well-known for various pharmacological activities, but most studies have been focused on red ginseng water extract (RGW), which has resulted in the conception of the present study of red ginseng oil (RGO) against Aβ_25–35_-induced neurotoxicity. Cytotoxicity and apoptosis induction by Aβ were verified and the underlying mechanism by which RGO inhibited neuronal cell death, mitochondria dysfunction and NF-κB pathway related protein markers were evaluated. RGO attenuated Aβ_25–35_-induced apoptosis, not only by inhibiting calcium influx, but also by reducing mitochondrial membrane potential loss. RGO significantly decreased Bax, whereas increased Bcl-2 and inactivated of caspase-3 and -9 and PARP-1 stimulated by Aβ_25–35_. Anti-neuroinflammatory effect of RGO was demonstrated by downregulating c-Jun N-terminal kinase (JNK) and p38, resulting in inhibiting of the NF-κB pathway and thereby suppressing the expressions of pro-inflammatory mediators such as inducible nitric oxide synthase (iNOS), cyclooxygenase-2 (COX-2), prostaglandin E_2_ (PGE_2_), nitric oxide (NO) and tumor necrosis factor-α (TNF-α). The present study revealed that RGO is a potential natural resource of the functional foods industry as well as a promising candidate of multi-target neuronal protective agent for the prevention of AD.

## 1. Introduction

Alzheimer’s disease (AD) is the most common form of dementia and is characterized by memory loss and cognitive impairment. The neuropathological hallmarks of AD are the presence of extracellular amyloid β protein (Aβ) deposits in senile plaques and intracellular composition of hyperphosphorylated tau proteins in neurofibrillary tangles (NFTs) [1]. The deposition of Aβ, derived from amyloid precursor protein (APP) after enzymatic cleavage by β-secretase (BACE1) and γ-secretase, is a key feature and is the trigger mechanism for AD [2].

Mitochondria play critical parts in the maintenance of cellular calcium homeostasis and the regulation of the cellular redox state. Studies have shown that Aβ_25–35_ could cause mitochondrial dysfunctions, such as glucose metabolism deficiency, the deactivation of key enzymes required for oxidative phosphorylation, and the accumulation of mitochondrial reactive free radicals [3]. Bcl-2, which belongs to anti-apoptotic Bcl-2 family, acts as a main regulator in the mitochondrial apoptosis pathway. The overexpression of Bcl-2 defends neurons from various neurotoxic damages. On the other hand, Bax, an important member of pro-apoptosis family, triggers apoptosis via translocation into the mitochondrial membrane and the subsequent facilitation of cytochrome c release [4]. Once released into the cytosol, cytochrome c interacts with Apaf-1 forming apoptosome, which initiates the switch of pro-caspase-9 to caspase-3 and ultimately leads to apoptosis [5]. In addition, overexpression of poly (ADP-ribose) polymerase-1 (PARP-1), which is involved in DNA repair, results in neurodegeneration [6]. PARP-1 is cleaved by the activation of caspase-3, which induces subsequently apoptosis and neuronal death.

The mechanism by which Aβ triggers an inflammatory processes is complex, but there is evidence that the peptide provokes the activation of the transcription factor NF-κB [7]. The NFκB transcription factor family consists of five members called p50, p52, p65 (RelA), c-Rel and RelB, all of which can form various homo- and heterodimers. NF-κB, which is the most commonly found in the cytoplasm as a heterodimer, is composed of p65 and p50 subunits, with inhibitory proteins known as IκBs. In response to inflammatory stimuli, IκB kinase (IKK) phosphorylates inhibitor of kappa Bs (IκBs) and free NF-κB is translocated to the nucleus binding to κB binding sites in the promoter regions of target genes. This leads to the transcription of pro-inflammatory mediators including inducible nitric oxide synthase (iNOS), cyclooxygenase-2 (COX-2), tumor necrosis factor-α (TNF-α), interleukin-1β (IL-1β) and etc., which further accelerate the secretion of nitric oxide (NO) and prostaglandin E_2_ (PGE_2_) [8]. In addition, mitogen-activated protein kinases (MAPKs) are engaged in the regulation of the production of key inflammatory mediators. Upon exposure to Aβ stimuli, c-Jun N-terminal kinase (JNK), extracellular signal–regulated kinase (ERK), and p38 MAPK are activated by phosphorylation at two sites (threonine and tyrosine residues), which controls the activation of NF-κB signaling pathway [9].

Ginseng has been used in Asia as a traditional medicine and a dietary supplement for more than 2000 years. There exist about 12 recognized species of ginseng depending on the method of classification. The well-known species of ginseng includes *Panax ginseng* C.A. Meyer, *Panax quinquefolius* L., *Panax japonicus* C.A. Meyer, and *Panax notoginseng* etc. Among them, *Panax ginseng* Meyer, known as Korean ginseng, is known to be effective in diabetes, hypertension, nociception, and cancer [10]. Several studies have revealed the protective effects of Korean red ginseng (KRG) extract on central nervous system. Tu et al. [11] suggested that KRG extract exerted neuroprotective effects on tau phosphorylation in human neuroblastoma cells. Moreover, KRG exhibited anti-apoptotic activity against oxidative stress in H_2_O_2_-stimulated SK-N-SH cells through the enhancement of Bcl-2 expression and the supression of caspase-3 activity [12]. It has been suggested that KRG extract can protect neurons against kainic acid-stimulated injury both in vitro and in vivo through antioxidant effect [13]. Moreover, the oral administration of KRG restored scopolamine-induced memory impairment in mice [14]. Nevertheless, these various beneficial properties of red ginseng were mainly reported for its water soluble extract, because red ginseng is mostly consumed as water extract or concentrate. Recently, red ginseng oil (RGO) has been studied for potential pharmacologically important roles. Various biological effects of RGO have been reported, including anticancer activity in hepatoma and breast cancer cell proliferation in vitro [15] and antioxidant and hepatoprotective effects in H_2_O_2_-treated HepG2 cells and CCl_4_-treated mice [16]. In our preliminary study, we reported the chemical profile and neuroprotective effect of RGO [17,18], which led to our interest in the mechanistic study of the RGO-mediated suppression of Aβ_25–35_-stimulated neurotoxicity through the oxidative and inflammatory signaling pathways. Therefore, the aim of the present study was to elucidate neuroprotective effects of RGO, and to provide a comparison of the effects with those of red ginseng water extract (RGW), against Aβ_25–35_-induced oxidative stress and neuroinflammation.

## 2. Results

### 2.1. RGO on Aβ_25–35_-Stimulated PC12 Cell Death

**Figure 1:**
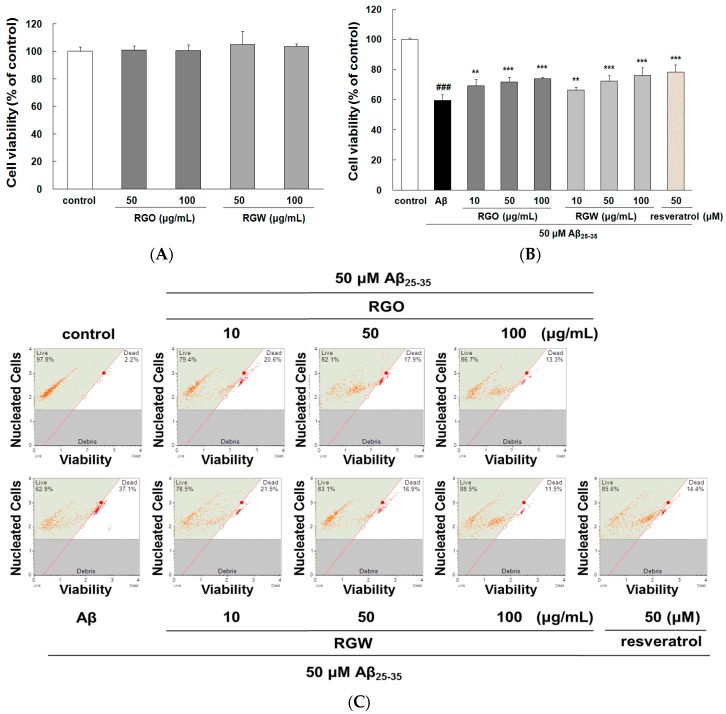

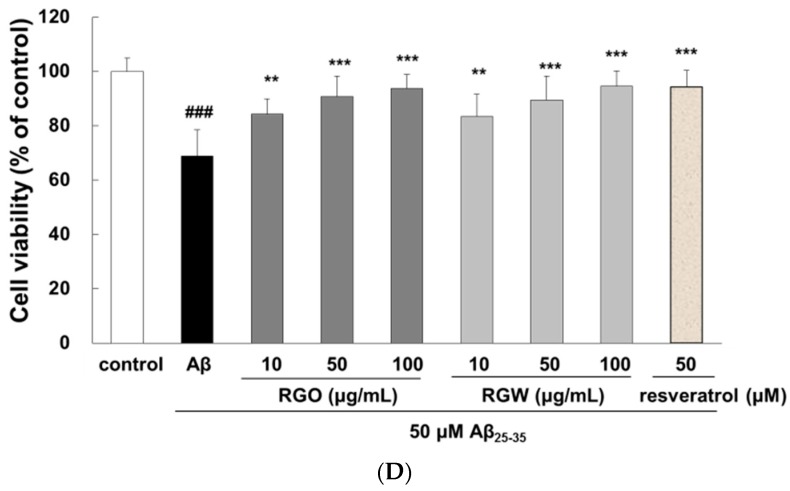
Red ginseng oil (RGO) and red ginseng water (RGW) protected Aβ_25–35_-stimulated cellular death. Assessment of cytotoxicity of RGO or RGW alone in PC12 cells (**A**). PC12 cells were treated with RGO or RGW for 1 h followed by exposure to 50 μM Aβ_25–35_ for 24 h, and cell viability was detected by 3-(4,5-dimethylthiazol-2-yl)-2,5-diphenyltetrazolium bromide (MTT) reduction assay (**B**) and flow cytometry (**C**). Statistical analysis results of the percentage of cell viability by flow cytometry (**D**). ### *p* < 0.001 compared to control group; *** *p* < 0.001 and ** *p* < 0.01 compared to Aβ_25–35_ alone (ANOVA followed by Duncan’s multiple comparison test). Resveratrol was used as a positive control.

As shown in Figure 1A, RGO and RGW did not affect cell viability even at the highest concentration up to 100 μg/mL. Moreover, single oral administration RGO (5 g/kg) to Sprague–Dawley rats induced no changes in cell viability [19]. Both results suggested that RGO and RGW could be safely used in further experiments as they were not cytotoxic. When stimulated with 50 μM Aβ_25–35_, cell viability markedly decreased to 59.57 ± 4.01% of that of the control group (Figure 1B), but the Aβ_25–35_-generated cytotoxicity was attenuated by treatment with RGO, as well as RGW. These results showed that both RGO and RGW had neuroprotective components.

An automated cell count and cell viability analysis was performed using double DNA intercalating fluorescent dyes by the Muse cell analyzer (Figure 1C,D). Aβ_25–35_treatment (50 μM) decreased PC12 cell viability to 68.8 ± 9.6% (*p* < 0.001), but this was substantially increased to 84.4 ± 5.5%, 90.8 ± 7.4%, and 93.8 ± 5.1% by pretreatment with RGO at 10, 50, and 100 μg/mL, respectively. RGW pretreatment (50 and 100 μg/mL) also increased the cell viability to 89.5 ± 8.8% and 94.5 ± 5.6%, respectively. The results indicated that RGO had a similar cytoprotective effect in Aβ_25–35_-damaged PC12 cells as that of RGW.

### 2.2. RGO on Aβ_25–35_-Stimulated Intracellular Reactive Oxygen Species (ROS) and Apoptosis

**Figure 2:**
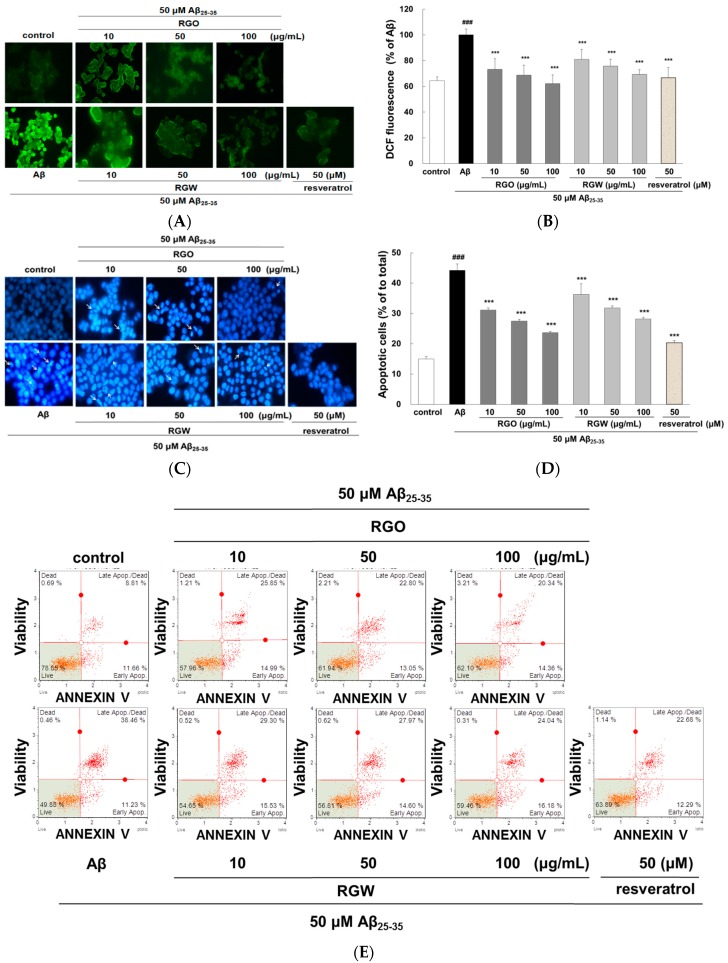

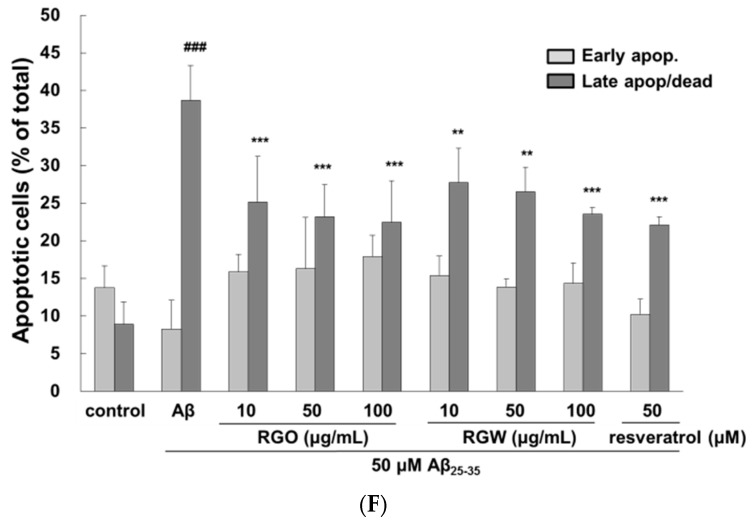
RGO and RGW inhibited Aβ_25–35_-stimulated intracellular reactive oxygen species (ROS) and apoptosis. PC12 cells were treated with samples for 1 h (10, 50, and 100 μg/mL) and further added with Aβ_25–35_ for 24 h. ROS generation of PC12 cells observed with CM-H2DCFDA (×400) (**A**) and measured by microplate reader (**B**). PC12 cell apoptosis was stained with Hoechst 33342 and imaged by fluorescence microscopy (**C**). Quantification of apoptotic cell (**D**). The flow cytometry images of apoptosis observed with Annexin V-7AAD (**E**). Percentage of early and late apoptotic cells (**F**). ### *p* < 0.001 compared to control group; *** *p* < 0.001 and ** *p* < 0.01 compared to Aβ_25–35_ alone (ANOVA followed by Duncan’s multiple comparison test). Resveratrol was used as a positive control.

The intracellular ROS scavenging ability of RGO was measured by fluorescent CM-H2DCFDA. The microscopy images clearly showed that the increased fluorescence in Aβ_25–35_-damaged PC12 cells was markedly reduced by RGO and RGW (Figure 2A). As presented in Figure 2B, Aβ_25–35_ stimulation of PC12 cells generated significantly higher level of ROS (100 ± 1.75%, *p* < 0.001) than that of the control (62.67 ± 2.39%). The results showed that both RGO and RGW treatment significantly decreased ROS production in a dose-dependent mode. In particular, RGO at 100 μg/mL reduced ROS generation similar the level of control group in the absence of Aβ_25–35_.

To measure the inhibitory property of RGO against Aβ_25–35_-stimulated apoptosis, PC12 cells were dyed with Hoechst 33342 and viewed under a fluorescence microscope (Figure 2C). Aβ_25–35_-stimulated apoptosis of PC12 cells was characterized by marked chromatin condensation. However, after pretreatment of RGO or RGW in addition of Aβ_25–35_, the number of apoptotic cells clearly decreased. As shown in Figure 2D, 50 μM Aβ_25–35_ increased the incidence of cellular apoptosis to 43.11 ± 1.64% in comparison with the control group (14.64 ± 0.73%), whereas pretreatment with RGO decreased the incidence of apoptosis to 31.10 ± 0.67%, 27.50 ± 0.54%, and 23.68 ± 0.43% at 10, 50, and 100 μg/mL, respectively. RGW reduced the rate of apoptosis to 45.39 ± 2.08%, 36.25 ± 3.53%, and 31.75 ± 0.84% at 10, 50, and 100 μg/mL, respectively. The ratio of early and late apoptotic cells was evaluated using flow cytometry after annexin V and 7-AAD staining [20]. The percentage of late apoptosis cells in the control sample was 8.9 ± 2.9%, whereas Aβ_25–35_ treatment significantly increased this to 38.7 ± 4.6% (*p* < 0.001). The increase in the late apoptotic cells was decreased by treatment with RGO, particularly at 10, 50, and 100 μM (to 25.1 ± 6.1%, 23.2 ± 6.1%, and 22.4 ± 5.6%, respectively). RGW also decreased the proportion of cells late apoptosis at 10, 50 and 100 μM (27.7 ± 4.6, 26.5 ± 3.2%, 23.5 ± 0.9%, respectively; Figure 2E,F).

The oxidative stress generated by Aβ causes apoptosis in neuronal cells [21]. The increase in apoptotic cells could result from the marked increase in ROS level; however, RGO attenuated this elevation. Especially, the inhibitory effect of RGO 100 μM was similar to that of resveratrol, a positive control without significant differences. The above results indicated that its dual roles as an antioxidant and anti-apoptotic agent in Aβ_25–35_-induced damage.

### 2.3. RGO on Aβ_25–35_-Stimulated Cell Cycle Changes and Mitochondrial Membrane Potential (MMP)

**Figure 3:**
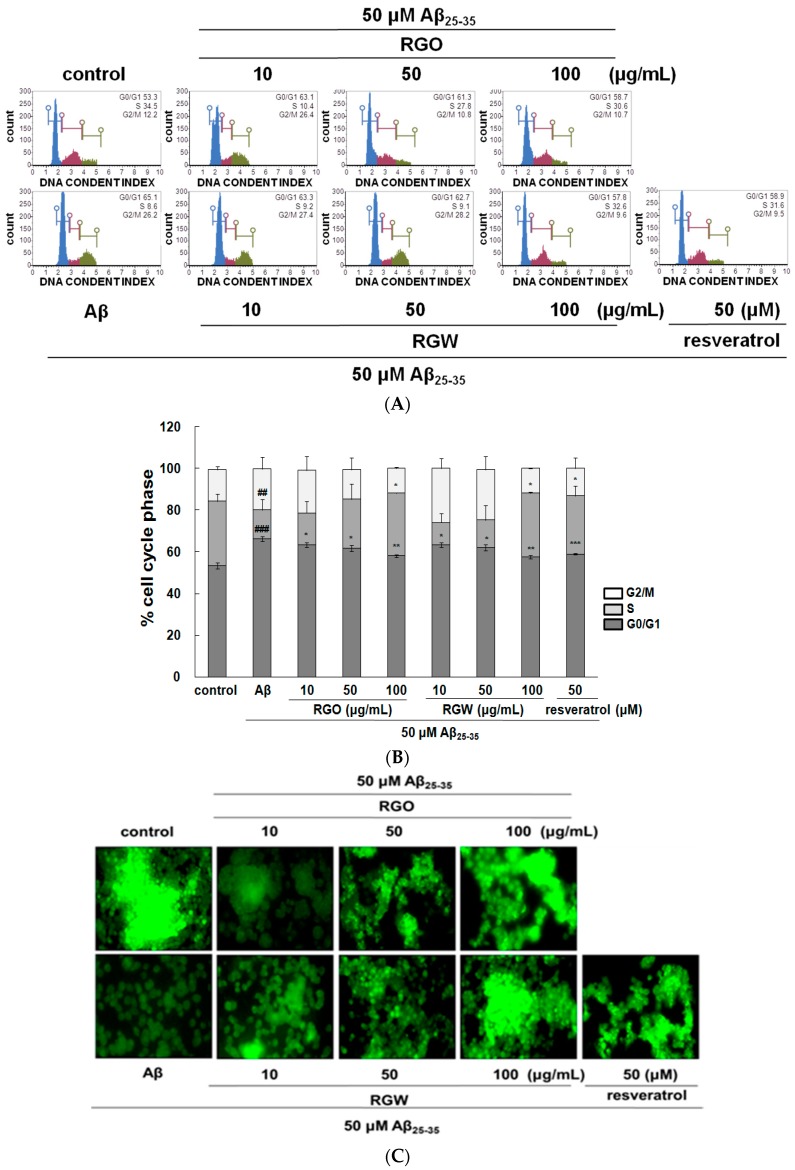

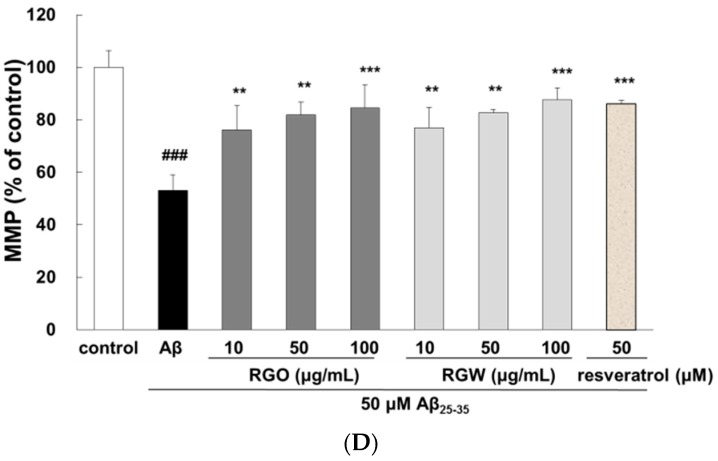
Effect of RGO and RGW on cell cycle and mitochondrial membrane potential (MMP). Flow cytometry of cell cycle phase (**A**) and quantification (**B**). Representative flow cytometry of Rhodamine 123 staining for MMP (×400) (**C**). Statistical analysis results of the percentage of MMP through Rhodamine 123 intensity (**D**). The graph shows the cell cycle and MMP as described in Materials and Methods. ### *p* < 0.001 and ## *p* < 0.01 compared to control group; *** *p* < 0.001, ** *p* < 0.01 and * *p* < 0.05 compared to Aβ_25–35_ alone (ANOVA followed by Duncan’s multiple comparison test). Resveratrol was used as a positive control.

Recent research indicated that Aβ provoked cell cycle change in the G0/G1 phase in senescent retinal pigment epithelial cells [22]. The analysis of PC12 cell cycle distribution after exposure to Aβ_25–35_ showed that the cellular population in G0/G1 phase increased from 53.5 ± 1.4% to 66.4 ± 1.2% and the S phase population decreased from 30.8 ± 1.37% to 13.6 ± 1.16%, which indicated that the cell cycle of Aβ_25–35_-induced PC12 cells was halted at G0/G1 phase. After RGO treatment, PC12 cells in the G0/G1 phase decreased and the cells in S phase increased. Lipid-soluble ginseng extract was reported to regulate cell cycle arrest in human lung cancer cells [23]. RGO, as well as RGW treatment, attenuated cell cycle arrest in Aβ_25–35_-treated PC12 cells and the highest concentrations of RGO and RGW almost restored the cells to the control values (Figure 3A,B).

The depolarization of mitochondria is considered an initial and irreversible step of apoptosis. To evaluate this, the MMP was determined by the evaluation of rhodamine fluorescence [24]. After Aβ_25–35_ exposure, MMP of PC12 cells was significantly reduced to 53.1 ± 5.9% compared with that of the control (100 ± 6.3%). However, the MMP was markedly recovered in the cells pretreated with RGO or RGW (100 μg/mL) by up to 84.4 ± 9.0% or 87.6 ± 4.6%, respectively (Figure 3C,D).

### 2.4. RGO on Aβ_25–35_-Induced Ca^2+^ Influx

**Figure 4:**
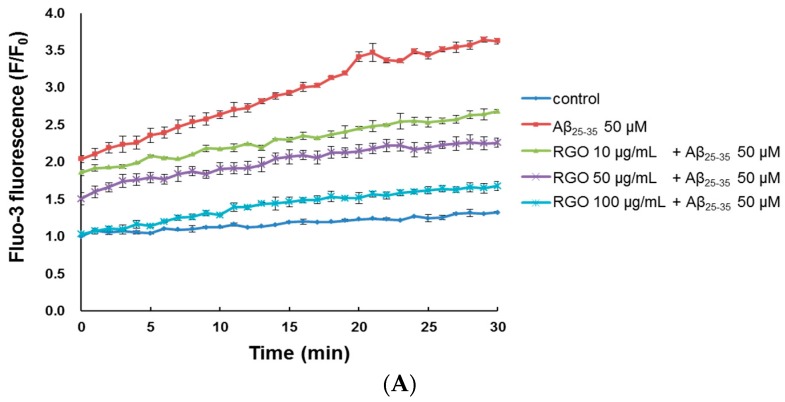

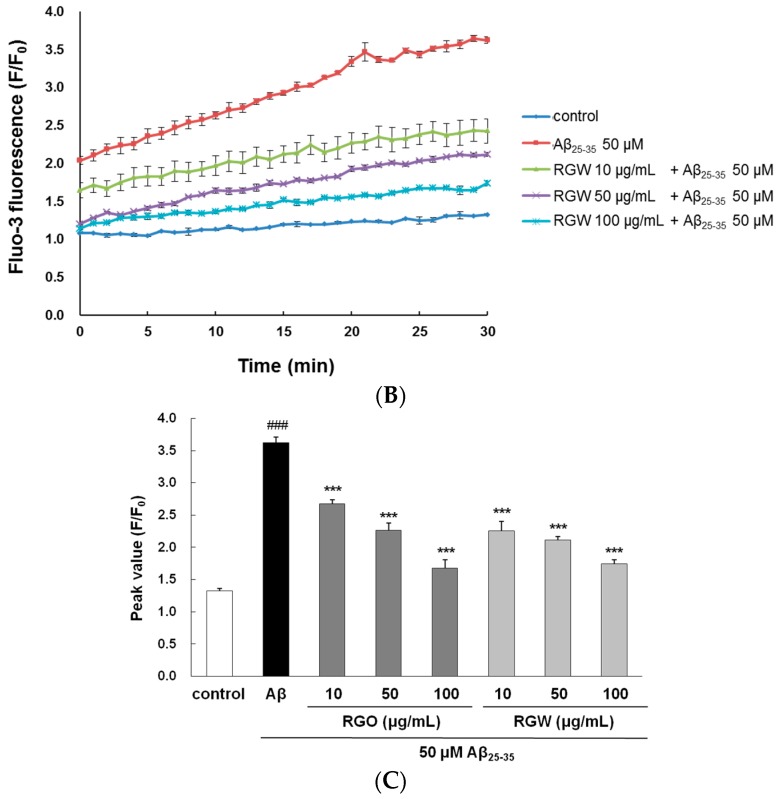
RGO (**A**) and RGW (**B**) ameliorated Aβ_25–35_-induced intracellular calcium levels ([Ca^2+^]i). Histogram showing the peak values of [Ca^2+^]i (**C**). The graph indicates calcium levels the as described in Materials and Methods. ### *p* < 0.001 compared to control group; *** *p* < 0.001 compared to Aβ_25–35_ alone (ANOVA followed by Duncan’s multiple comparison test).

Cytoplasmic Ca^2+^ level is a vital regulator of neurotransmitter and neural development; Ca^2+^ dysregulation, particularly overload, results in neuronal cell death [25]. The level of cytosolic free Ca^2+^ was measured through evaluation of a fluorescent Ca^2+^ indicator, Fluo-3/AM, after a 30-min treatment. Aβ_25–35_-treatment increased cytosolic free Ca^2+^, producing a peak level of 3.62 ± 0.08 in comparison with the control peak level of 1.33 ± 0.03 (Figure 4A–C). However, treatment with RGO or RGW significantly suppressed the increase in cytosolic free Ca^2+^ level (*p* < 0.001). 

### 2.5. RGO on Aβ_25–35_-Stimulated Changes in Bcl-2 and Bax Expression

**Figure 5:**
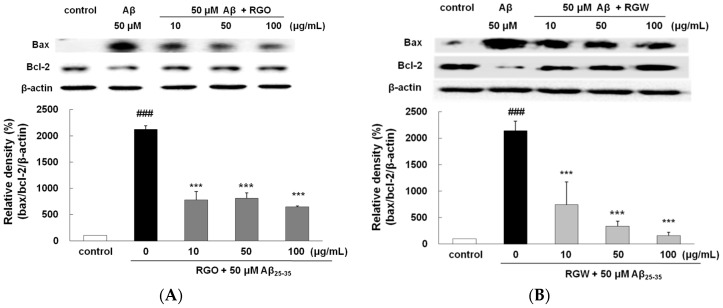

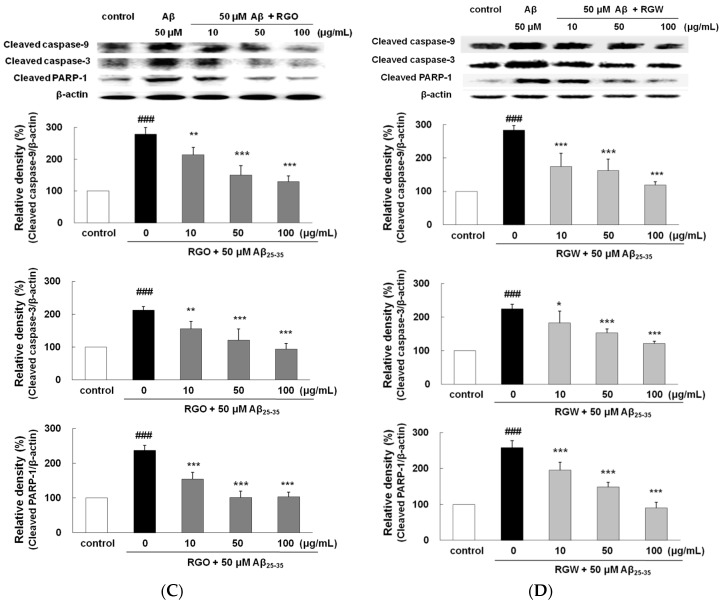
RGO (**A**) and RGW (**B**) inhibited Aβ_25–35_-induced Bax/Bcl-2 expression. Bax and Bcl-2 expression levels were normalized against the expression levels of the loading control, β-actin. RGO (**C**) or RGW (**D**) blocked Aβ_25–35_-induced protein levels of cleaved caspase-3 (19 kDa, Y shape), cleaved caspase-9 (35 kDa, Y shape) and cleaved PARP-1 (89 kDa, Y shape) in PC12 cells. PC12 cells were treated with Aβ_25–35_ for 24 h and then cellular lysates were analyzed by Western blot assay. The separated proteins were analyzed by Western blot analysis as described in Materials and Methods. ### *p <* 0.001 compared to control group; *** *p* < 0.001, ** *p* < 0.01 and * *p* < 0.05 compared to Aβ_25–35_ alone (ANOVA followed by Duncan’s multiple comparison test).

It is well established that the ratio of Bax/Bcl-2 plays an important role in mitochondria- mediated apoptosis [26]. The Bax/Bcl-2 ratio significantly elevated in Aβ_25–35_ group (Figure 5A,B). In comparison with that in the Aβ_25–35_ group, the expression of Bax decreased in the RGO and RGW groups, whereas the expression of Bcl-2 increased at 10, 50, and 100 μg/mL. The incidence of apoptosis in the RGO- or RGW-treated samples was significantly lower at all the concentrations tested in comparison with that of the Aβ_25–35_ group.

### 2.6. RGO on Aβ_25–35_-Stimulated Expression of Caspase-3, Caspase-9, and PARP-1

Western blot analysis exhibited that PARP-1 cleavage in Aβ_25–35_-treated PC12 cells resulted in enhanced caspase-3 activity, which occurred during apoptosis and neurodegeneration. The levels of both caspase-9 and caspase-3 significantly increased with Aβ_25–35_-stimulaion. Pretreatment with RGO or RGW resulted in the amelioration of Aβ_25–35_-induced neurotoxicity and dose-dependently suppressed caspase-9 and caspase-3 level in comparison with that observed with Aβ_25–35_-treatment (Figure 5C,D). Similarly, the levels of PARP-1 increased in Aβ_25–35_-stimulated PC12 cells, of which was reduced by pretreatment with RGO or RGW. In particular, all concentrations of RGO showed a significant inhibitory effect on caspase-9, caspase-3, and PARP-1.

These results corresponded with those observed in Aβ_25–35_-induced primary cultured rat cortical cells. The aqueous extract from red ginseng was reported to exert neuroprotective property through inhibition of apoptosis-related molecules including Bax, caspase-3, and PARP-1, in addition to reduced BACE1 activity [27]. The present study affirmed the effects and mechanisms of action of RGO in comparison with that of RGW against Aβ-induced oxidative and inflammatory damage in PC12 cells for the first time.

### 2.7. RGO on Aβ_25–35_-Stimulated TNF-α Production

**Figure 6:**
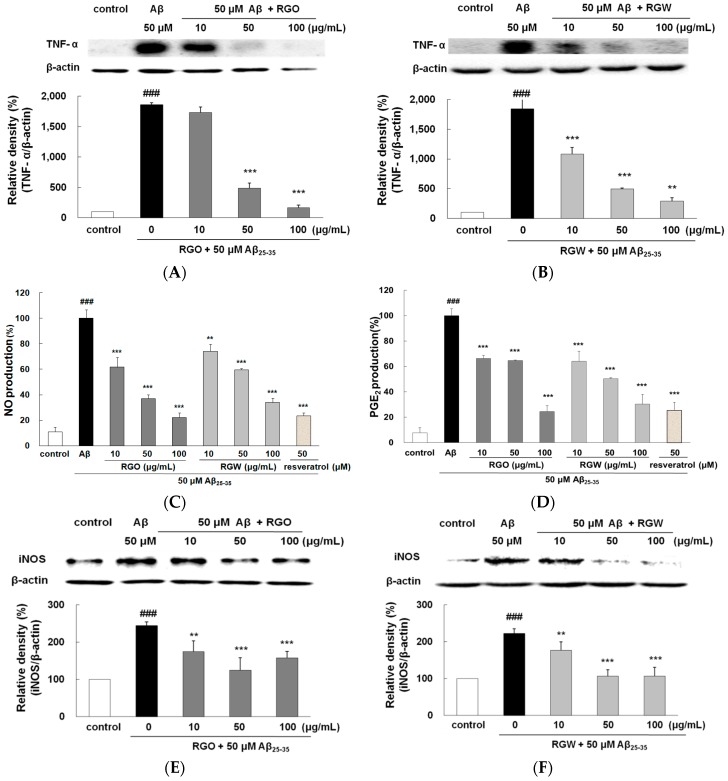

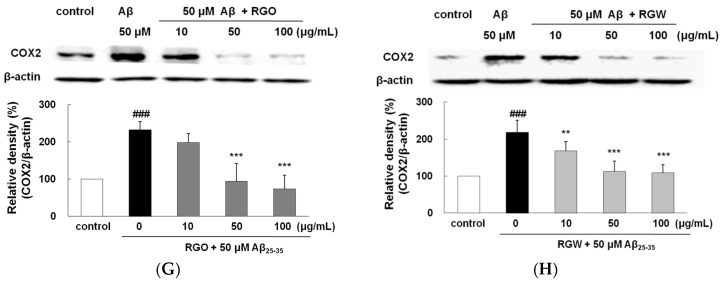
RGO (**A**) and RGW (**B**) protected Aβ_25–35_-stimulated TNF-α production. PC12 cells were treated with Aβ_25–35_ for 30 h and then cellular lysates were analyzed by Western blot assay. Cells were pretreated with RGO or RGW for 1 h, then stimulated with 50 μM Aβ_25–35_. The culture medium samples were collected 24 h later for NO and PGE_2_ assay. Effect of RGO and RGW on production of NO (**C**) and PGE_2_ (**D**). Effect of RGO on expression of iNOS (**E**) and COX-2 (**G**). Effect of RGW on expression of iNOS (**F**) and COX-2 (**H**). PC12 cells were treated with Aβ_25–35_ for 30 h and then cellular lysates were analyzed by Western blot assay. The separated proteins were analyzed by Western blot, Griess and ELISA assay as described in Materials and Methods. ### *p* < 0.001 compared to control group; *** *p* < 0.001 and ** *p* < 0.01 compared to Aβ_25–35_ alone (ANOVA followed by Duncan’s multiple comparison test).

TNF-α is recognized to promote cell survival and death in the central nervous system. Immunohistochemical studies have revealed an increase in TNF-α localized to senile plaques, which suggested its participation in Aβ-induced inflammation [8]. As shown in Figure 6A,B, the release of TNF-α was significantly higher in Aβ_25–35_-treated PC12 cells than in the untreated control (*p* < 0.001). The TNF-α levels were dose-dependently suppressed by pretreatment with RGO and RGW. In particular, when the cells were preincubated with 100 μg/mL RGO, TNF-α production was markedly suppressed to a similar value as that of the unstimulated control (*p* < 0.001).

### 2.8. RGO on Aβ_25–35_-Stimulated NO/PGE_2_ Production and iNOS/COX-2 Expression

Aβ_25–35_-treatment exhibited a marked NO and PGE_2_ increase (approximately 10-fold) compared with the control group (Figure 6C,D). With the presence of RGO, NO and PGE_2_ production significantly decreased. Western blot analysis demonstrated that Aβ_25–35_ markedly increased both iNOS and COX-2 level. However, RGO effectively blocked the expression of iNOS (Figure 6E,F) and caused the dose-dependent downregulation of the Aβ_25–35_-stimulated COX-2 expression (Figure 6G,H). NO plays a key part in various tissues at normal physiological concentration. However, abnormal high level of NO generated by iNOS has been revealed as a cause of inflammatory cytotoxicity [28,29].

### 2.9. RGO on Aβ_25–35_-Stimulated NF-κB and IκB-α Phosphorylation

**Figure 7:**
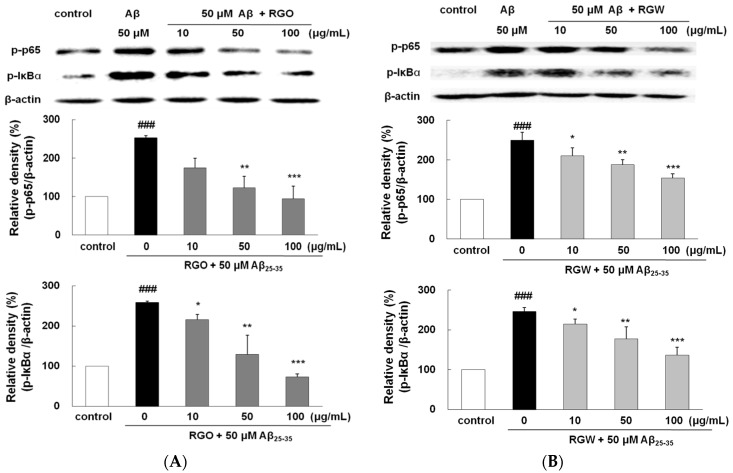
RGO (**A**) and RGW (**B**) blocked Aβ_25–35_-stimulated NF-κB and IκBα expression. PC12 cells were treated with Aβ_25–35_ for 4 h and then cellular lysates were analyzed by Western blot assay. The separated proteins were analyzed by Western blot analysis as described in Materials and Methods. ### *p* < 0.001 compared to control group; *** *p* < 0.001, ** *p* < 0.01 and * *p* < 0.05 compared to Aβ_25–35_ alone (ANOVA followed by Duncan’s multiple comparison test).

Aβ triggers the activation of NF-κB, leading to gene transcription responsible for the inflammatory responses accompanying Aβ neurotoxicity [30]. Aβ_25–35_ increased p65 level significantly by 252.2 ± 5.8%. However, both RGO effectively blocked the phosphorylation of p65 (Figure 7A,B). In particular, RGO at 100 μg/mL resulted in almost complete inhibition of p65 phosphorylation (*p* < 0.01). Aβ_25–35_-triggered IκB-α phosphorylation was impeded by RGO pretreatment. Unexpectedly, RGO potently showed much better suppression in Aβ_25–35_-triggered IκB-α phosphorylation than RGW, which clearly implied that RGO exerted its anti-inflammatory action via the suppression of both p65 and IκB-α activation.

### 2.10. RGO on Aβ_25–35_-Induced MAPK Activation

**Figure 8:**
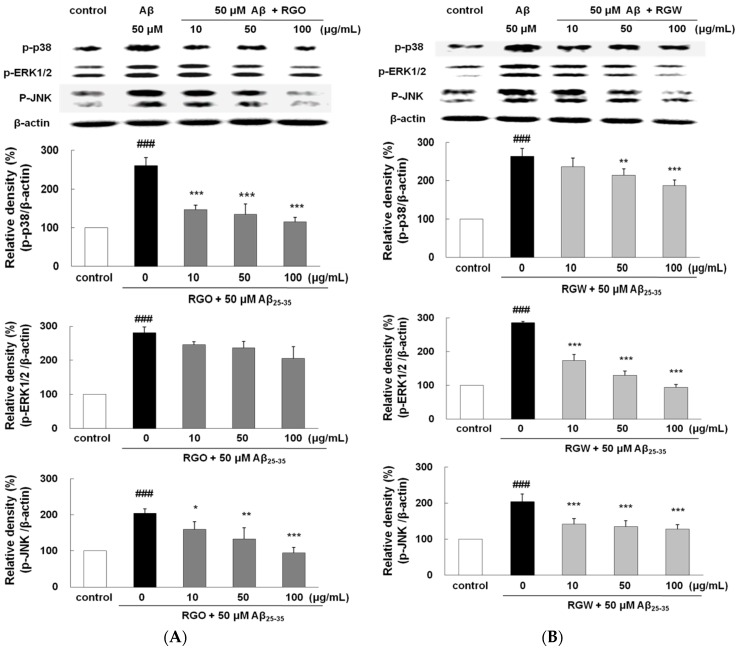
RGO (**A**) and RGW (**B**) ameliorated Aβ_25–35_-stimulated MAPKs phosphorylation. PC12 cells were treated with Aβ_25–35_ for 1 h and then cellular lysates were analyzed by Western blot assay. The separated proteins were analyzed by Western blot analysis as described in Materials and Methods. ### *p* < 0.001 compared to control group; *** *p* < 0.001, ** *p* < 0.01 and * *p* < 0.05 compared to Aβ_25–35_ alone (ANOVA followed by Duncan’s multiple comparison test).

To examine whether the suppression of NF-κB by RGO or RGW was provoked via MAPK pathway, Aβ_25–35_-stimulated phosphorylation of MAPKs was analyzed. After Aβ_25–35_ treatment, the phosphorylation levels of p38, ERK1/2, and JNK significantly elevated (Figure 8A,B). Although RGO did not affect ERK1/2, it notably attenuated the Aβ_25–35_-stimulated p38 and JNK phosphorylation; in particular, p38 was downregulated to 123.8 ± 6.8%, 123.4 ± 14.4%, and 107.7 ± 17.6% at 10, 50, and 100 μg/mL, respectively. In contrast, the suppression of JNK and p38 by RGW was relatively weak compared with that of RGO; however, RGW pretreatment strongly attenuated the Aβ_25–35_-induced phosphorylation of ERK1/2 in a dose-dependent manner. Overall, our results demonstrated that the inhibitory mechanisms of NF-κB activation by RGO and RGW were different.

## 3. Discussion

RGW has already been known to protect SK-N-SH cells, hippocampal cells and primary cultured rat cortical cells against H_2_O_2_-induced oxidative stress, kainate and Aβ-induced neurotoxicity, respectively, implying its additional beneficial actions in the CNS [12,13]. The present study showed that RGW, as well as RGO, mitigated the toxicity of Aβ_25–35_ via regulation of ROS, apoptosis and pro-inflammatory mediator. RGO and RGW could mediate both extrinsic and intrinsic apoptosis pathways with regulation of not only MMP, intracellular calcium, Bax/Bcl-2 ratio but also caspase-9, -3 and -8. Furthermore, all extracts inhibited iNOS and COX-2 expression through suppression of NF-κB and IκB. It is noteworthy that both RGO and RGW at high concentration, though in the form of extract, exhibited similar or better neuroprotective property than that of resveratrol, a positive compound.

In terms of inflammation, American ginseng attenuated iNOS expression in LPS-stimulated RAW 264.7 cells [31]. Another study reported that *Panax quinquefolius* suppressed the expression of iNOS and COX-2 in colitic mice [30]. RGO also exerted anti-inflammatory effects through inhibition of COX-2 and iNOS in RAW264.7 macrophages stimulated with LPS [32]. The strong inhibition of NF-κB may occur through the modulation of p38 and JNK by RGO; in contrast, RGW may modulate ERK1/2, JNK, and p-38. Similar results have been reported for RGO treatment in LPS-induced RAW264.7 macrophages was related with the inhibition of NF-κB transcriptional activity, possibly through blocking of the p38 MAPK pathway [32].

Much attention has been paid to lipid-soluble bioactive compounds and/or essential oils from plants that exhibit greater bioavailability than water-soluble compounds. Several plant-derived essential oils such as *Tornabenea bischoffiii* and *Zataria multiflora* were reported to possess antioxidant properties via radical scavenging and antioxidant enzymes induction [33,34]. In addition, the effects of essential oils on pathological targets of AD such as Aβ deposition, NFTs, cholinergic hypofunction, oxidative stress and glutamatergic abnormalities were focused [35].

Miroddi et al. (2014) systematically and extensively reviewed the clinical trials assessing pharmacological properties of *Salvia* species (sage) including *S. officinalis* and *S. lavandulaefolia* on memory, cognitive impairment and AD [36]. Sage treatment including *S. lavandulaefolia* essential oil and sunflower oil for total 6 weeks as enhanced memory, reduced neuropsychiatric symptoms and improved in attention [37,38]. The majority of potentially bioactive hydrocarbons contained in *S. lavandulaefolia* are including acyclic, monocyclic, bicyclic compounds, diterpenes and triterpenes. *Nigella sativa*, possessing the active constituent thymoquinone, significantly decreased the number of hippocampal cells death and decreased lipid peroxidation induced by iron ascorbate in hippocampal homogenate [39].

Farr et al. (2012) revealed the effects of extra virgin olive oils on learning and memory in SAMP8 mice, an age-related learning/memory impairment model associated with increased Aβ and brain oxidative damage [40]. Administration of extra virgin olive oils to a transgenic mouse model of AD-like TgSwDI, reduced parenchymal and vascular Aβ levels via an enhancement of its clearance [41]. In addition, extra virgin olive oils-rich diet in 3xTg mice model had an amelioration of their behavioral deficits, increase in the steady state levels of synaptophysin, a protein marker of synaptic integrity, reduction in insoluble Aβ levels and deposition, lower amount of phosphorylated tau protein at specific epitopes [42]. Furthermore, more than 30 compounds including oleocanthal and oeluropein aglycone are demonstrated to possess such beneficial effects against Aβ and tau pathologies in AD (Farr et al., 2012) [40].

In a high-fat diet-treated obese mouse, the administration of fresh ginseng oil of suppressed TG absorption and/or regulated PPAR-γ expression [43]. RGW, which consists predominantly of ginsenosides and acidic polysaccharides, has been the subject of various biological investigations. In contrast, the composition and content of RGO vary depending on the method of extraction and the parts of red ginseng used. GC-MS analysis revealed that the hexane extract of red ginseng contained panaxynol, a type of ginseng polyacetylene [44]. Our previous study suggested that the major compounds of RGO after supercritical fluid extraction were linoleic acid (31.48%), bicyclo[10.1.0]tridec-1-ene (22.54%), β-sitosterol, (16.90%), ergosta-4,6,22-trien-3α-ol (4.53%), and γ-sitosterol (4.31%) [17]. The analysis of the fatty acid composition of red ginseng extracted with petroleum ether also indicated that linoleic acid was the most prevalent component [45]. Lee et al. [46] reported that red ginseng seed oil contained the highest content of oleic acid (80%). Recently, some studies demonstrated neuroprotective effects of linoleic acid, oleic acid, and β-sitosterol [18,47,48,49]. Although there were positive correlations between lipid-soluble components and neuroprotective properties in the present study, it was unclear whether the neuroprotective activity of RGO resulted from its major constituent compounds. Therefore, further studies are required to investigate in vitro and in vivo neuroprotective effects of the active compounds of RGO.

## 4. Materials and Methods

### 4.1. Materials

Roswell Park Memorial Institute (RPMI) cell culture medium, FBS, PBS, donor equine serum, trypsin-EDTA and penicillin-streptomycin were obtained from Hyclone Laboratories (Logan, UT, USA). HBSS N2 supplement, fluo-3/AM, CM-H2DCFDA, Hoechst 33342, and pluronic F-127 were purchased from Invitrogen (Carlsbad, CA, USA). Aβ_25–35_ (synthetic, ≥97% HPLC), MTT, rhodamine123, and resveratrol as a positive control were obtained from Sigma-Aldrich (St. Louis, MO, USA). Specific antibodies and other cell-culture relating assay kits were purchased according to the previous studies [4,45]. All organic solvents were analytical grade. Antibodies against iNOS, COX-2, TNF-α, cleaved-caspase-9, cleaved-PARP-1, bax, bcl-2, β-actin, monoclonal antibodies, and peroxidase-conjugated secondary antibody were purchased from Santa Cruz Biotechnology Inc. (Santa Cruz, CA, USA). Anti-phospho-JNK, phospho-ERK, phospho-p38, phospho-IκB-α, phospho-p65, and cleaved-caspase-3 monoclonal antibodies were purchased from Cell Signaling Technology Inc. (Beverly, MA, USA).

### 4.2. Preparation of Samples

RGO was prepared as previously described with a slight modification [16]. Red ginseng powder (6-year old) was purchased from the Korea Ginseng Corporation (Cheong-Kwan-Jang, Seoul, Korea). For RGO, dried red ginseng powder (200 g) was extracted using the supercritical CO_2_ extraction system at pressure of 6500 psi (450 bar) and 65 °C (Ilshin Autoclave Co. Ltd., Daejeon, Korea). The oil extract was collected in a vial that was prefilled with trapping solvent and maintained at 4 °C. The water extraction of red ginseng was performed using a slight modification of previously described procedure [50]. For RGW, red ginseng powder (10 g) was extracted with hot water (200 mL) heated for 1 h at 90 °C in a water bath (Daihan Scientific, Seoul, Korea). After cooling, the supernatant was collected by centrifugation (3000× *g*) and evaporated. After extraction, the samples were stored at 4 °C until used and no more than 3 days prior to experiments.

### 4.3. Cell Culture and Peptides

PC12 cells obtained from the American Type Culture Collection (ATCC, Manassas, VA, USA), maintaining in RPMI 1640 supplemented with 5% FBS, 10% horse serum, and 100 U/mL penicillin, were grown in a humidified atmosphere of 5% CO_2_ at 37 °C. For inducing aggregation for toxicity, Aβ_25–35_ in PBS was incubated at 37 °C for 48 h [37].

### 4.4. Cell Viability

Cultured PC12 cells were pretreated with RGO or RGW (10–100 μg/mL) or vehicle only for 1 h and then exposed to 50 μM preaggregated Aβ_25–35_ for another 24 h in the continued presence of sample or vehicle. MTT solution (5 mg/mL of stock in PBS) was added and incubated for 3 h. The absorbance was read at 570 nm using ELX808 microplate reader (BioTek, Winooski, VT, USA). 

### 4.5. Intracellular ROS Accumulation

Intracellular ROS level of was monitored using fluorescent probe of DCF. PC12 cells were stained with CM-H_2_DCFDA (10 μM) at 37 °C in the dark for 30 min and resuspended in HBSS. Cells were observed by fluorescence microscope (Olympus, Tokyo, Japan), and the fluorescent intensity of ROS was detected with excitation and emission at 485 and 528 nm, respectively, using microplate reader (FLX800, Winooski, VT, USA).

### 4.6. Fluorescent Staining of Cells with Hoechst 33342

The apoptosis was measured by Hoechst 33342 fluorescent dye. PC12 cells were fixed using 4% formaldehyde and then incubated with 50 μM Hoechst 33342 for 15 min. The nuclear morphology was observed with fluorescence microscope by manual. 

### 4.7. MMP Assay

PC12 cells were incubated with 5 μM rhodamine 123 (final concentration) at 37 °C for 30 min. After incubation, cells were rinsed with PBS, followed by visualization under a microscope (Olympus, Tokyo, Japan). The fluorescent intensity was detected by a FLX800 microplate reader with excitation and emission at 485 nm and 530 nm, respectively.

### 4.8. Intracellular Free Calcium

Quantitative intracellular calcium was evaluated using fluo-3/AM, fluorescence indicator of free calcium. Cultured cells were incubated with fluo-3/AM and pluronic F-127 for 30 min at 37 °C. After washing HBS, fluorescence was measured in the FLX800 plate reader (485 nm excitation, 528 nm emission) [25].

### 4.9. Nitrite Determination

Cell culture media (100 μL) were mixed with equal volume of Greiss reagent in a 96-well plate. After 10 min incubation at room temperature, nitrite production was calculated from the absorbance of the mixture at 550 nm using standard curve of NaNO_2_.

### 4.10. PGE_2_ Enzyme-Linked Immunoassay

The medium and primary antibody solution were added to a 96-well plate which was pre-coated with goat anti-mouse IgG and then PGE_2_ conjugate was added. After 1 h reaction, any unbound antibody-enzyme reagent was washed and removed. Substrate solution was added and the intensity (450 nm) was detected using ELX808 microplate reader.

### 4.11. Flow Cytometry Analysis and Western Blot Analysis

Cell viability, cell cycle and apoptosis were measured using Muse™ Cell Analyzer (Millipore, Billerica, MA, USA). All the reagents were utilized according to the manufacturer’s guidelines [37]. Western blot analysis were performed according to the previous studies [4,37] and the density of Western blot bands was analyzed using Atto EZ-capture ST Imaging software (Tokyo, Japan).

### 4.12. Statistical Analysis

All data were expressed as mean ± SD derived from more than three independent experiments. All the data were initially checked for normality using the PROC univariate procedure in SAS version 9.3 (SAS Institute, Cary, NC, USA). Statistical comparisons were conducted using one-way analysis of variance (ANOVA) followed by Duncan’s post hoc multiple comparison test using SAS. 

## 5. Conclusions

This study offered the first indication that RGO inhibited Aβ_25–35_-stimulated neuronal apoptosis and inflammation in PC12 cells. RGO significantly alleviated mitochondrial dysfunction through an improvement in the MMP, the recovery of Bcl-2/Bax ratio, and the regulation of mitochondria-mediated apoptosis by the inactivation of caspase-3, caspase-9, and PARP-1. In addition, anti-neuroinflammatory property of RGO have been exerted through the downregulation of p38 and JNK, which resulted in the inhibition of NF-κB pathway and thus suppressed the expression of pro-inflammatory mediators including COX-2, iNOS, PGE_2_, NO, and TNF-α. Collectively, these results supported the potential for the development of RGO as a protective treatment for Aβ-induced cellular damage in the AD process, although the in vivo effects of RGO require further investigation.
